# Editorial: Intelligent Recognition and Detection in Neuroimaging

**DOI:** 10.3389/fnins.2022.943180

**Published:** 2022-07-05

**Authors:** Yu-Dong Zhang, German Castellanos-Dominguez, Yuankai Huo, Juan Manuel Gorriz, Roohallah Alizadehsani

**Affiliations:** ^1^School of Computing and Mathematical Sciences, University of Leicester, Leicester, United Kingdom; ^2^Department of Electrical Engineering, National University of Colombia, Manizales, Colombia; ^3^Department of Electrical Engineering and Computer Science, Vanderbilt University, Nashville, TN, United States; ^4^Department of Signal Theory, Networking and Communications, University of Granada, Granada, Spain; ^5^Institute for Intelligent Systems Research and Innovation, Deakin University, Geelong, VIC, Australia

**Keywords:** intelligent recognition, neuroimaging, EEG, MRI, PET

Neuroimaging scan uses various techniques to either directly or indirectly image the nervous system's structure, function, or pharmacology. Those scans are being used more and more to help detect and diagnose many medical disorders and illnesses. Currently, brain scans for mental disorders are in research studies to learn more about the disorders. Many neuroimaging methods are used in hospitals and research institutes, such as computed tomography, event-related optical imaging, magnetic resonance imaging (MRI), functional MRI, positron emission tomography, single-photon emission computed tomography, magnetoencephalography, etc. Brain scans alone are commonly used to diagnose neurological and psychiatric diseases, such as Meningioma, glioma, Herpes encephalitis, Huntington's disease, Pick's disease, Alzheimer's disease, Multiple sclerosis, and cerebral palsy toxoplasmosis, Sarcoma, Subdural hematoma, etc.

Intelligent recognition and detection use the latest pattern recognition (PR) methods to recognize and detect suspicious areas of neuroimaging scan images. Also, we can realize the analysis, enhancement, reconstruction, segmentation, and classification of those neuroimaging scan images. Those PR methods include support vector machine, deep learning, transfer learning, convolutional neural network, graph neural network, attention neural network, explainable AI, trustworthy AI, etc. This intelligent recognition and detection are still an ongoing developing field. Hence, we expect our topic can intrigue more fascinating works in this area.

This topic was online, calling for papers from April/2021. It received more than 40 submissions from over 30 different countries. After strict peer reviews, only eight papers are accepted and published. All the papers are research articles.

## Voice Data

Voice data is a meaningful way to help diagnose diseases. Ali et al. investigated Parkinson's disease (PD). Multimodal voice data are put into two channels, i.e., the Smart Phone (SP) and Acoustic Cardioid (AC). Four types of data modalities are collected through each channel, namely sustained phonation (P), speech (S), voiced (V), and unvoiced (U) modality. The contributions of this paper are two-fold. First, it explores optimal data modality and features with better PD information. Second, it proposes a MultiModal Data-Driven Ensemble (MMDD-Ensemble) approach for PD detection. Experimental results show that their proposed multimodal approach yields 96% accuracy, 100% sensitivity, 88.88% specificity, 0.914 MCC, and 0.986 AUC. Their results are promising compared to the recently reported results for PD detection based on multimodal voice data.

## EEG

Electroencephalography (EEG) is a method to record an electrogram of electrical activity. Shoeibi et al. provide various intelligent deep learning (DL)-based methods for automated Schizophrenia (SZ) diagnosis *via* EEG signals. The obtained results are compared with those of conventional intelligent methods. The Institute of Psychiatry and Neurology dataset in Warsaw, Poland, has been used. First, EEG signals are divided into 25 s time frames and normalized by z-score or norm L2. In the classification step, two approaches are considered for SZ diagnosis *via* EEG signals. In this step, the classification of EEG signals is first carried out by conventional machine learning methods, e.g., support vector machine, k-nearest neighbors, decision tree, naïve Bayes, random forest, extremely randomized trees, and bagging. Various proposed DL models, namely, long short-term memories (LSTMs), one-dimensional convolutional networks (1D-CNN s), and 1D-CNN-LSTMs, are used. The DL models are implemented and compared with different activation functions in this step. Among the proposed DL models, the CNN-LSTM architecture has the best performance. Their proposed CNN-LSTM model has achieved an accuracy percentage of 99.25%, better than the results of most former studies in this field.

## Retina Images

Diabetic retinopathy (DR) is one of the common chronic complications of diabetes and the most common blinding eye disease. Ai, Huang, Fan, et al. propose an algorithm for detecting diabetic retinopathy based on deep ensemble learning and attention mechanism. First, image samples are preprocessed and enhanced to obtain high-quality image data. Second, to improve the adaptability and accuracy of the detection algorithm, the authors construct a holistic detection model DR-IIXRN, which consists of Inception V3, InceptionResNet V2, Xception, ResNeXt101, and NASNetLarge. For each base classifier, the authors modify the network model using transfer learning, fine-tuning, and attention mechanisms to improve its ability to detect DR. Finally, a weighted voting algorithm is used to determine which category (normal, mild, moderate, severe, or proliferative DR) the images belonged to. The authors also tune the trained network model on the hospital data, and the real test samples in the hospital also confirm the advantages of the algorithm in detecting the diabetic retina. Experiments show that compared with the traditional single network model detection algorithm, the AUC, accuracy, and recall rate of their proposed method are improved to 95, 92, and 92%, respectively.

## MRI

Magnetic resonance imaging (MRI) is a medical imaging technique used in radiology to form pictures of the anatomy of the body. Xiao et al. propose a new approach named “TReC: Transferred Residual Networks (ResNet)-Convolutional Block Attention Module (CBAM)”, a specific model for small-scale samples to detect brain diseases based on MRI. At first, the ResNet model, which is pre-trained on the ImageNet dataset, serves as initialization. Subsequently, a simple attention mechanism named CBAM is introduced and added to every ResNet residual block. At the same time, the fully connected (FC) layers of the ResNet are replaced with new FC layers, which meet the goal of classification. Finally, all the parameters of this model, such as the ResNet, the CBAM, and new FC layers, are retrained. Their model achieves accuracy results of 100% for the two-class task and 97.44% for the multi-class task. Compared with other state-of-the-art models, their model reaches the best performance.

Zhu et al. propose three novel models to classify brain diseases to cope with these problems. The proposed models are DenseNet-based SNN (DSNN), DenseNet-based RVFL (DRVFL), and DenseNet-based ELM (DELM). The backbone of the three proposed models is the pre-trained “customized” DenseNet. The modified DenseNet is fine-tuned on the empirical data set. Finally, the last five layers of the fine-tuned DenseNet are substituted by SNN, ELM, and RVFL, respectively. The accuracy, sensitivity, specificity, precision, and F1-score of their proposed DSNN on the test set are 98.46 ± 2.05%, 100.00 ± 0.00%, 85.00 ± 20.00%, 98.36 ± 2.17%, and 99.16 ± 1.11%, respectively. The proposed DSNN is compared with restricted DenseNet, spiking neural network, and other state-of-the-art methods. Overall, the DSNN gets the best performance among the three proposed models in classification performance.

Meng et al. propose a novel voxel-based feature detection framework for AD. Specifically, using 649 voxel-based morphometry (VBM) methods obtained from MRI in Alzheimer's Disease Neuroimaging Initiative (ADNI), the authors propose a feature detection method according to the Random Survey Support Vector Machines (RS-SVM) and combined the research process based on image-, gene-, and pathway-level analysis for AD prediction. Remarkably, the authors constructed 136, 141, and 113 novel voxel-based features for EMCI (early mild cognitive impairment)-HC (healthy control), LMCI (late mild cognitive impairment)-HC, and AD-HC groups, respectively. The authors applied the linear regression model, least absolute shrinkage and selection operator (Lasso), partial least squares (PLS), SVM, and RS-SVM five methods to test and compare the accuracy of these features in these three groups. The prediction accuracy of the AD-HC group using the RS-SVM method was higher than 90%. The experimental results using five machine learning indicate that the identified features are effective for AD and HC classification, the RS-SVM framework has the best classification accuracy, and their strategy can identify important brain regions for AD.

## PET

Positron emission tomography (PET) is a functional imaging technique that uses radioactive substances known as radiotracers to visualize and measure changes in metabolic processes. Jin et al. propose a hybrid deep learning method. First, a classification network is pretrained with paired MRI and PET images. Afterward, the authors use the pretrained classification network to guide a GAN by focusing on the helpful features for classification. Finally, the authors synthesize the missing PET images and use them with real MR images to fine-tune the classification model to make it better adapt to the synthesized images. The authors evaluate the proposed method on the ADNI dataset, and the results show that the proposed method improves the accuracies obtained on the validation and testing sets by 3.84 and 5.82%, respectively. Moreover, the proposed method increases the accuracies for the validation and testing sets by 7.7 and 9.09%, respectively, when the authors synthesize the missing PET images *via* our method.

## Optical Coherence Tomography

Optical coherence tomography (OCT) is a new type of tomography that has experienced rapid development and potential. Ai, Huang, Feng, et al. propose a fusion network (FN)-based retinal OCT classification algorithm to improve the adaptability and accuracy of traditional classification algorithms. The InceptionV3, Inception-ResNet, and Xception deep learning algorithms are used as base classifiers, a convolutional block attention mechanism (CBAM) is added after each base classifier, and three different fusion strategies are used to merge the prediction results of the base classifiers to output the final prediction results. The results show that in a classification problem involving the UCSD common retinal OCT dataset (108,312 OCT images from 4,686 patients), compared with the InceptionV3 network model, the prediction accuracy of FN-OCT is improved by 5.3% (accuracy = 98.7%, AUC = 99.1%). The predictive accuracy and AUC achieved on an external dataset for the classification of retinal OCT diseases are 92 and 94.5%, respectively.

## Conclusion

This topic reports the recent intelligent recognition and detection methods/applications *via* voice data, EEG, retina images, MRI, PET, and OCT. With the help of intelligent recognition and detection methods, those models can help realize analysis, enhancement, reconstruction, segmentation, and classification. Some of these models yield better performances than state-of-the-art. Remarkably, some models are explainable, giving visual heat maps of the location of regions of interest.

We expect this Research Topic will provide a comprehensive picture of recent intelligent recognition and detection methods using artificial intelligence and deep learning. This Research Topic will serve as a starting point for designing more complex intelligent recognition and detection methods, which may be applied in medical image analysis fields.

## Author Contributions

All authors listed have made a substantial, direct, and intellectual contribution to the work and approved it for publication.

## Conflict of Interest

The authors declare that the research was conducted in the absence of any commercial or financial relationships that could be construed as a potential conflict of interest.

## Publisher's Note

All claims expressed in this article are solely those of the authors and do not necessarily represent those of their affiliated organizations, or those of the publisher, the editors and the reviewers. Any product that may be evaluated in this article, or claim that may be made by its manufacturer, is not guaranteed or endorsed by the publisher.

